# Genome Wide Identification of *LIM* Genes in *Cicer arietinum* and Response of *Ca-2LIMs* in Development, Hormone and Pathogenic Stress

**DOI:** 10.1371/journal.pone.0138719

**Published:** 2015-09-29

**Authors:** Vikas Srivastava, Praveen Kumar Verma

**Affiliations:** Plant Immunity Laboratory, National Institute of Plant Genome Research, Aruna Asaf Ali Marg, New Delhi, India; Institute of Botany, Chinese Academy of Sciences, CHINA

## Abstract

The eukaryotic lineage-specific LIM protein (*L* *IN11*, *I* *SL1*, and *M* *EC3*) family play pivotal role in modulation of actin dynamics and transcriptional regulation. The systematic investigation of this family has not been carried in detail and rare in legumes. Current study involves the mining of *Cicer arietinum* genome for the genes coding for LIM domain proteins and displayed significant homology with LIM genes of other species. The analysis led to the identification of 15 members, which were positioned on chickpea chromosomes. The phylogenetic and motif analysis suggested their categorization into two sub-families *i*.*e*., Ca-2LIMs and Ca-DA1/DAR, which comprised of nine and six candidates, respectively. Further sub-categories of *Ca-2LIMs* were recognised as αLIM, βLIM, δLIM and γLIM. The LIM genes within their sub-families displayed conserved genomic and motif organization. The expression pattern of *Ca-2LIMs* across developmental and reproductive tissues demonstrated strong correlation with established consensus. The *Ca-2LIM* belongs to *PLIM* and *GLIM (XLIM)* was found highly expressed in floral tissue. Others showed ubiquitous expression pattern with their dominance in stem. Under hormonal and pathogenic conditions these LIMs were found to up-regulate during salicylic acid, abscisic acid and *Ascochyta rabiei* treatment or infection; and down-regulated in response to jasmonic acid treatment. The findings of this work, particularly in terms of modulation of LIM genes under biotic stress will open up the way to further explore and establish the role of chickpea LIMs in plant defense response.

## Introduction

Transcription factors are one of the most fundamental attractions for the modulation of various biological processes. The features like morphological, developmental and biosynthetic or even in combat against various stresses are modulated by them [1–3]. The easy accessibility of high throughput genome sequencing technologies has facilitated many researchers to carry out sequencing of whole genome. The recent outlook is to identify individual gene(s) and subsequent functional study on entire gene family to elucidate their precise role in various biological events. The LIM family proteins are one of the important ubiquitous transcription factor known in eukaryotic lineage. This family named after epithet of three initially discovered LIM homeo-domain proteins (*L* *IN11*, *I* *SL1*, and *M* *EC3*). These proteins comprised of a novel cysteine-rich zinc-binding domain which has been described in mammals, amphibians, flies, worms and plants. The LIM domain functions as a protein–protein interaction module, with the consensus sequence [C-X_2_-C-X_16–23_-H-X_2_-C]-X_2_-[C-X_2_-C-X_16–21_C-X_2–3_-(C/D/H)] and essentially comprised of two zinc fingers linked together by a short two-amino acid spacer [4]. The gene coding for plant LIM domain containing proteins are of two sub-families. The first is similar to Cysteine Rich Proteins (CRPs) of animals that include two LIM domains separated by a long inter-LIM domain, with some differences like long C-terminal and absence of glycine rich regions (GRR) [5]. However, the second (DA1 and DAR) comprises UIM (Ubiquitin Interaction Motifs) and DUF3633 as characteristic domain in addition to single LIM domain [6]. DAR can be separated from DA proteins due to the absence of UIM [7].

Diverse role of these proteins in various cellular processes, including construction of cytoskeleton, transcription control and context-dependent development have been portrayed [8–10]. Though this protein has been illustrated in all eukaryotic cells long back, but the functional relevance of this gene family in plants are very low and need much attention. The first LIM identified as SF3 for *Helianthus* later re-named as HaPLIM1 was found specifically in pollen grains [11]. Since then, the LIM domain containing proteins has been identified in *Arabidopsis*, *Populus*, *Oryza*, *Nicotiana*, *Eucalyptus*, *Gossypium*, *Glycine* and *Brassica* [5,6,8,12–15]. Nearly a decade from its discovery, this protein family has been commonly considered as a player having role in the regulation of actin dynamics, which is also an established fact in animals. The first evidence of its impact on transcriptional modulation was noticed to regulate key genes of phenyl-propanoid pathway *viz*., *CAD*, *PAL* and *4CL* by NtLIM1 [8]. Furthermore, the dual function of these proteins was explored recently in *Nicotiana* and *Gossypium*, where the LIM domain protein functions in both ways [13,16]. Additionally, new role of two LIM domain containing proteins in the regulation of histone activation was also observed [16]. Though the findings regarding the transcriptional regulation are being reported from number of plant systems, but its utility in this way needs special attention. No such function of LIMs was noticed till date particularly in *Arabidopsis thaliana* and *Oryza sativa*. The comparative genome analysis of this gene family has been assessed in *A*. *thaliana* and *O*. *sativa*, which revealed six members in each. However, in *Populus trichocarpa* the number increased to 12 due to duplication [5]. The recent survey of *Brassica rapa*, *BrLIMs* showed 22 proteins with at least one LIM domain and comprised of 14 two LIM domain containing proteins and 8 DA1/DAR with single LIM domain along with additional domains [14].

Legumes have unique role in supplementing proteins to human diet in cheapest way and thus have a definite role to wipe out protein deficiency diseases and it is a major concern for most of the developing countries. Chickpea (*Cicer arietinum*) holds third position in the world as an imperative pulse crop cultivated and consumed throughout the globe [17]. Recently, the draft genome as well as *de novo* transcriptome assembly has been made available in public databases [18–20], which have promoted chickpea as an alternative legume crop to investigate gene families.

In the present study, the genes encoding LIM domain containing protein were identified from available chickpea genome databases [19–20] and revealed 15 putative candidates. The identified genes were named as per established consensus. The phylogenetic and motif analysis were carried out which revealed two well demarcated sub-families. Additionally, *in silico* expression profiles were analysed in various organs and developmental conditions using Chickpea Transcriptome Database (CTDB) (www.nipgr.res.in/ctdb.html). To further validate role in development, their expression was analysed by semi-quantitative RT-PCR using vegetative and reproductive tissues. Furthermore, their expression kinetics was also examined under hormonal (SA, JA and ABA treatment) and pathogenic (*Ascochyta rabiei* spore inoculation) conditions. Overall, the present investigation provide new insights over the responses of this important though less explored plant gene family and will laid prospects for functional characterization of individual LIM genes for the improvement of this important legume crop.

## Materials and Methods

### Identification of the *CaLIM* genes in chickpea

The sequences of *CaLIMs* in chickpea (CDC Frontier ‘Kabuli’ cultivar, Bioproject: PRJNA175619) were identified by TBLASTN analysis using known protein sequences of established *Arabidopsis* LIMs. The encoded proteins of predicted *CaLIMs* were then analysed individually by SMART and BLASTP analysis, to give confidence to prediction. The comparison of all the predicted sequences were also performed using the genome of small-seeded ‘desi’ chickpea ICC4958 cultivar (Bioproject: PRJNA78951; http://nipgr.res.in/CGAP). The Pfam-ID 00412 (LIM domain) and 12315 (DUF3633) was used as key word for the confirmation of putative members in ICC4958 cultivar.

### Mapping of *CaLIMs* on chickpea chromosomes and gene organization

The *CaLIM* genes of chickpea were searched for BLASTN analysis against available chickpea genome (CDC Frontier ‘Kabuli’ cultivar, Bioproject: PRJNA175619). The position of the genes was given in order of their appearance on chromosomes. The gene structure was obtained after alignment of individual LIM open reading frame with their respective gene on genome. The representation of the gene was performed by online Gene Structure Display Server (http://gsds.cbi.pku.edu.cn/).

### Sequence alignment, Phylogenetic, motif and protein analysis

The deduced LIM (2LIM + DA1/DAR) proteins of chickpea along with other plants (*B*. *rapa*, *O*. *sativa*, *A*. *thaliana*) retrieved from NCBI and published work [5,14] were aligned with PROMALS3D (http://prodata.swmed.edu/promals3d), a program based on multiple sequence and structure alignment [21]. For alignment of 2LIM proteins *P*. *trichocarpa* was also included along with others. The alignment of sixty-one LIMs and forty seven 2LIMs were used to generate phylogenetic tree using MEGA v.6.0 software [22]. Maximum Likelihood (ML) tree was constructed using JTT + G and JTT+G+I (after best method prediction) for LIMs and 2LIMs, respectively. ML tree was constructed for LIM proteins of chickpea using Dayhoff +G model. The ML tree was also made for DA1 proteins of chickpea and soybean using JTT +G model. The bootstraps were performed for 1000 iteration and partial deletion was used for gap treatment. The conserved motifs of all LIM proteins were carried out using MEME Suite (http://meme.nbcr.net/meme/cgi-bin/meme.cgi) with: any number of repetitions, minimum width of six amino acids, maximum width of 50 amino acids, and the maximum number of motifs up to 10. ProtParam analysis (http://web.expasy.org/protparam) was performed for each protein sequence to identify number of amino acid residues, molecular weight, pI value and GRAVY index. The prediction of subcellular localization was performed by WoLF PSORT v0.2 (http://www.genscript.com/psort/wolf_psort.html).

### *In silico* expression data using CTDB database

The expression data for available *CaLIM* genes were retrieved from the Chickpea Transcriptome Database (CTDB) (http://www.nipgr.res.in/ctdb.html). The data were used to generate heat map for various tissues and developmental stages. The description of sample collection and further processing for transcriptome analysis was earlier reported [23–24] and also referenced in detail [25].

### Plant materials, tissue collection, hormone treatment and *A*. *rabiei* infection

The seeds of *C*. *arietinum* L. (Pusa-362) were grown in phytotron (16 h light/ 8 h dark at 25°C) and in field of National Institute of Plant Genome Research (NIPGR), New Delhi. Different vegetative and reproductive tissues were collected after two months old field-grown plants. All the harvested tissues (roots, stem, leaves and flowers) were immediately frozen in liquid nitrogen and stored at −80°C till further experiments. The virulent *A*. *rabiei* (Delhi isolate ITCC No: 4638) cultures maintained in our laboratory were used for infection and all the other chemicals viz. salicylic acid (SA), jasmonic acid (JA) and abscisic acid (ABA) were purchased form Sigma, India. The 3 weeks old phytotron-grown plants were treated with plant defense hormones at the concentration of 5mM of SA, 100μM of JA and 100μM of ABA. The samples were collected as described above after 0, 0.5, 3, 12 and 24 h of spray of respective hormones. The spore suspension of freshly grown *A*. *rabiei* cultures (2 X 10° spores/mL) were sprayed onto 3 weeks old phytotron-grown plants and almost one-third of the aerial part of the respective samples were collected in triplicate after 0, 6, 12, 24 and 72 h. The confirmation of infection was also monitored on same lot by observing the appearance of lesion on further co-incubation. The collected samples from treated/infected were frozen immediately in liquid nitrogen and stored at -80°C until RNA isolation.

### RNA isolation and Real Time analysis

The RNA was extracted from various tissues and stress treated frozen samples using TRIzol reagent (Invitrogen). The isolated RNA was treated with RNase-free DNase (Promega, USA) to eliminate any contamination of genomic DNA. The quantity and purity of total RNA were assessed using NanoDrop ND-1000 Spectrophotometer (NanoDrop Technologies, Wilmington, DE, USA). The total RNA (1.2 μg) isolated from each samples was reverse transcribed into cDNA using the High Capacity cDNA Reverse Transcription Kits (Applied Biosystems, Foster City, CA, USA) and Oligo-dT primers. The Primer Express^®^ (version 3.0) software (Applied Biosystems) was used to design gene-specific primers for *Ca-2LIM* genes (S1 Table). The specificity of primer pair was visualized by dissociation curve monitoring and agarose gel electrophoresis. The qRT-PCR was performed using 7900 HT Fast Real-Time PCR System (Applied Biosystems, Foster City, CA). The reaction mixture comprised of 4.1 μl of DNase/RNase free water, 10 μl of Real-Time SYBR Green PCR master mix, 0.3 μL of ROX dye (50 times diluted), 2 μL diluted cDNA (100 times diluted) and 1.8 μL each of gene-specific primers (Agilent Technologies). The thermal cycle applied was as follows: 95°C for 3 min followed by 45 cycles of denature at 95°C for 5 s and annealing and elongation at 60°C for 15 s. The EF-1α and β-tubulin were used as internal reference for various tissues and treated samples, respectively. The relative fold analysis was performed in relation to roots and mock control for various tissues and treated samples, respectively. The 2^−ΔΔCt^ method was adopted to calculate relative gene expression [26] and each experiment were performed in triplicates. The heat map for gene expression patterns was generated with freely available online software Multi Experiment Viewer.

## Results

### Identification of genes encoding LIM domain in chickpea

**Fig 1:**
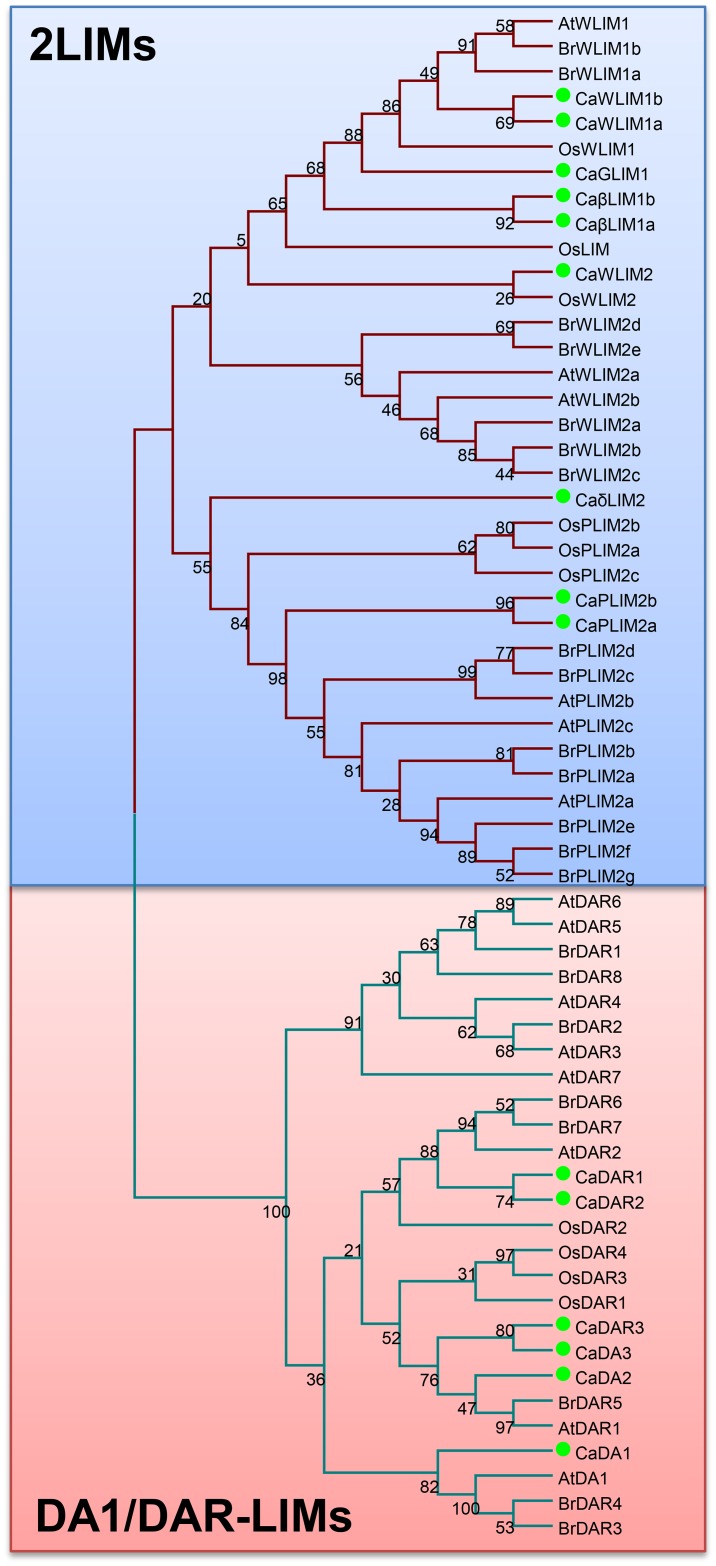
Phylogenetic tree demonstrating the evolutionary relation of the deduced full-length amino acid sequences of 15 CaLIMs with LIM proteins of *A*. *thaliana*, *B*. *rapa* and *O*. *sativa*. The unrooted phylogenetic tree was constructed using MEGA 6.0 by Maximum likelihood method with 1000 bootstraps. Bootstrap values are presented next to branch node. Green closed circles were used to show CaLIM proteins. Two major divisions were presented in different colours.

**Fig 2:**
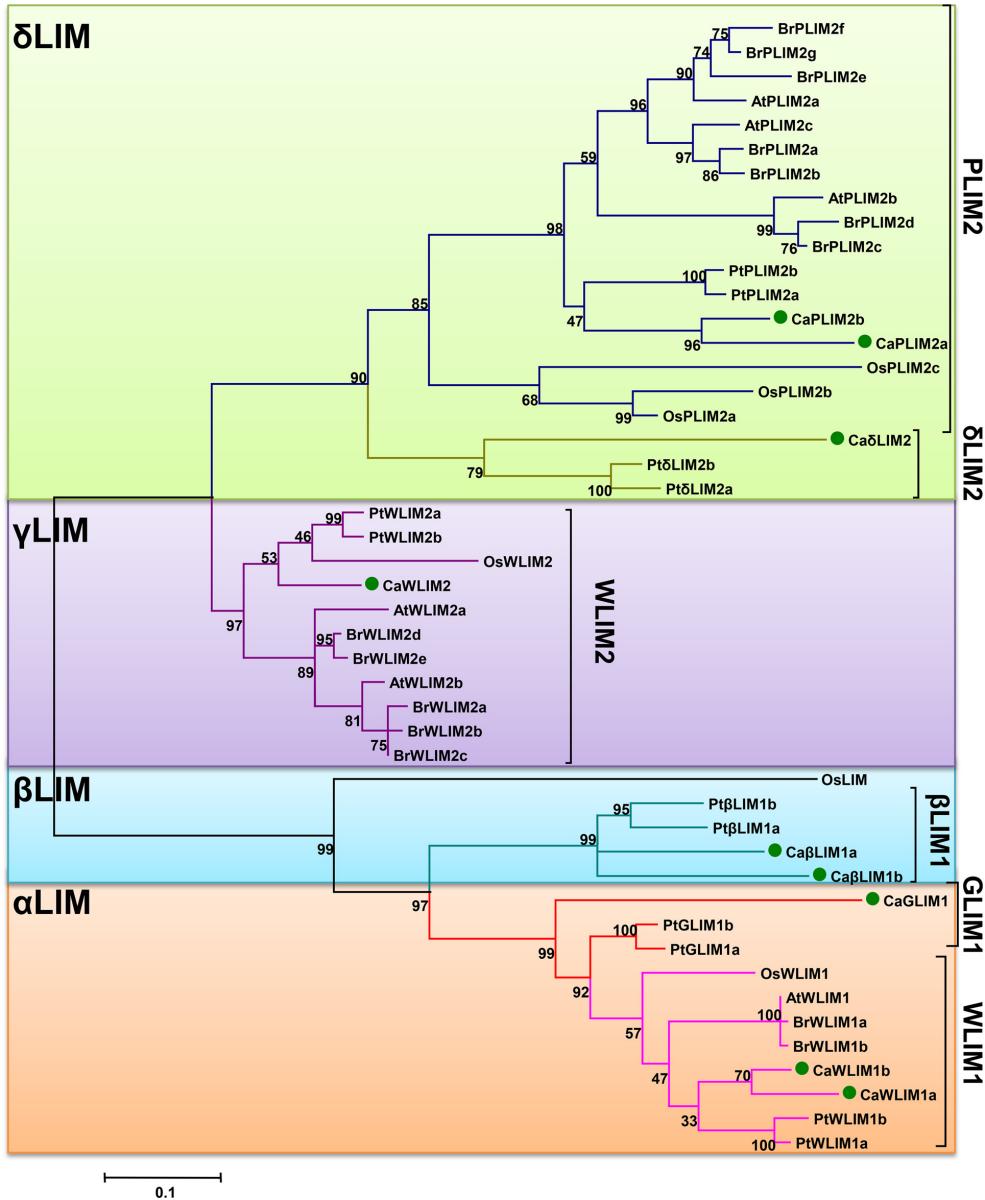
Phylogenetic tree demonstrating the evolutionary relation of the deduced full-length amino acid sequences of Ca-2LIMs with members of *A*. *thaliana*, *B*. *rapa*, *O*. *sativa* and *P*. *trichocarpa*. The unrooted phylogenetic tree was constructed using MEGA 6.0 by Maximum likelihood method with 1000 bootstraps. Bootstrap values are presented next to branch node. Different sub-groups are enclosed in separate coloured boxes.

**Fig 3:**
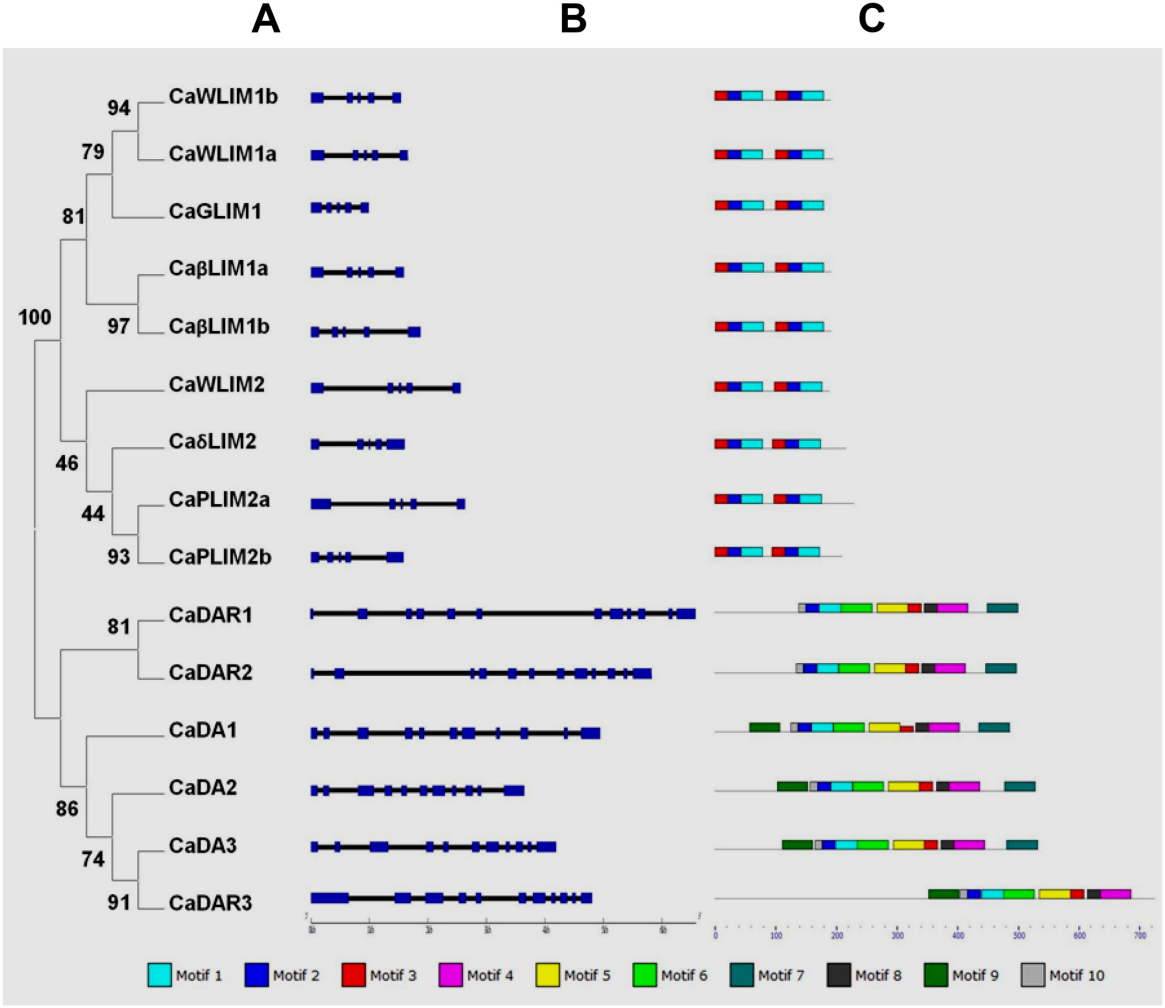
Phylogenetic analysis, gene structure and motif analysis of LIM family in *Cicer arietinum*. (A) Phylogenetic tree of CaLIMs was constructed using Maximum-likelihood method and the bootstrap test was performed for 1,000 repetitions. (B) The exon/intron organization of LIM genes of *C*. *arietinum*. Blue boxes represent exons and black lines indicate introns. (C) The conserved protein motifs in the LIM proteins identified using MEME program. Each motif is indicated with a specific color.

The BLAST search was performed against available chickpea genome and chickpea transcriptome database (CTDB) by using *A*. *thaliana* LIM domain-containing proteins. The chickpea LIM proteins were targeted using *A*. *thaliana* two LIM domain containing protein and DA1 protein as query sequence. The genome analysis indicated a total of 15 coding genes which code for at least a single LIM domain. For convenience, the genes encoding for these proteins were named as *CaLIM1* to *CaLIM15*, based on their location in the chickpea genome (Table 1). In order to verify the reliability of these sequences the deduced proteins were submitted for SMART analysis (http://smart.embl-heidelberg.de/), which showed the presence of LIM domain in each case (S1 Fig). The chickpea LIM proteins were grouped into two categories or sub-families (Fig 1). The first sub-family is represented by nine ORF similar to animal CRPs (Figs 2 and 3A). This group comprised of sequences ranges from 546–693 bp encoding 181–230 amino acids (Table 1). The second sub-family of proteins are similar to *Arabidopsis* DA1/DAR and represented by 6 members. This sub-family consist of single LIM domain along with DUF3633 at C-terminal and occasional presence of UIMs at N-terminal [14]. The *Ca-DA1/DAR* comprised of relatively longer sequences ranges from 1467–2178 bp encoding 488–725 amino acids (Table 1). The DAR members are separated from DA1 due to the absence of UIMs (S1 Fig). The depictions of individual domains are also visible in CLUSTALX2 based alignment (S2 Fig). Interestingly, the SMART analysis of predicted protein of *CaLIM14* exhibited additional domains at N-terminal such as RPW8 and NB-ARC. Here, the LIM domain also presents deviation from normal consensus due to absence of first cysteine residue. This gene was earlier annotated as pseudo-gene in available assembly (Genebank Project: PRJNA175619). The other features of deduced proteins such as molecular weight (20.5204 to 83.1475 kDa), pI (5.20 to 9.18) and GRAVY index (-0.777 to -0.426) were also presented. The WoLF-PSORT prediction suggested the localization of CaLIMs to either nucleus or cytoplasm or both (Table 1).

**Table 1:** Principal attributes of LIM gene members and their deduced proteins in *Cicer arietinum*.

Genes	CDC Frontier Contig ID (Contig. Assigned)	Gene ID	Chromosome Location	Name Assigned	Genomic Sequence (Bp)	ORF (Bp)	Protein					
							Length (aa)	LIM domain Start-end (aa)	Mol. Wt. (kDa)	pI	GRAVY	WoLF PSORT prediction
*CaLIM1*	ANPC01003576 (Ca2_1375)	LOC101512468	Chr2:29331256..29338189	*CaDAR1*	6580	1503	500	138–190	56.9183	7.11	-0.590	Nucl: 12, Cyto: 1
*CaLIM2*	ANPC01005127 (Ca3_1259)	LOC101497370	Chr3:22828873..22831800	*CaPLIM2a*	2630	693	230	9–61 & 106–158	26.1194	5.62	-0.777	Nucl: 5, Cyto: 5, Mito: 3
*CaLIM3*	ANPC01006169 (Ca4_473)	LOC101488743	Chr4:14511323..14512614	*CaGLIM1*	987	546	181	10–62 & 109–161	20.5204	8.87	-0.566	Chlo: 7, Mito: 3, Nucl: 2, Plas: 1
*CaLIM4*	ANPC01007681 (Ca4_1985)	LOC101511662	Chr4:45835305..45837390	*CaWLIM1a*	1657	588	195	9–61 & 109–161	21.7068	9.05	-0.596	Cyto: 6, Mito: 3, Nucl: 2, Chlo: 1, Plas: 1
*CaLIM5*	ANPC01007729 (Ca4_2033)	LOC101499377	Chr4:47443848..47449868	*CaDA1*	4939	1467	488	125–177	55.9081	6.27	-0.624	Nucl: 13
*CaLIM6*	ANPC01009522 (Ca5_1730)	LOC101499389	Chr5:36282869..36284924	*CaWLIM1b*	1532	576	191	9–61 & 109–161	21.2352	9.01	-0.619	Chlo: 8, Nucl: 4, Plas: 1
*CaLIM7*	ANPC01009653 (Ca5_1861)	LOC101505455	Chr5:39890308..39892781	*CaβLIM1a*	1584	579	192	10–62 & 109–161	21.8327	9.05	-0.619	Chlo: 9, Nucl: 3, Mito: 1
*CaLIM8*	ANPC01009971-72 (Ca6-71/72)	LOC101502367	Chr6:1608985..1615484	*CaDAR2*	5823	1491	497	134–186	56.6863	8.36	-0.582	Nucl: 8, Vacu: 2, Chlo: 1, Cyto: 1, Mito: 1
*CaLIM9*	ANPC01009974 (Ca6_74)	LOC101507830	Chr6:1815880..1819067	*CaWLIM2*	2554	570	189	9–61 & 107–159	20.8168	9.14	-0.492	Nucl: 10, Mito: 4
*CaLIM10*	ANPC01010074 (Ca6_174)	LOC101513290	Chr6:5687517..5689620	*CaδLIM2*	1600	654	217	9–61 & 104–156	23.7506	7.92	-0.588	Nucl: 8, Mito: 5
*CaLIM11*	ANPC01012830 (Ca7_350)	LOC101513735	Chr7:9381408..9383794	*CaβLIM1b*	1872	579	192	10–62 & 109–161	22.1023	9.18	-0.616	Chlo: 6, Nucl: 6, Mito: 1
*CaLIM12*	ANPC01012959 (Ca7_479)	LOC101505272	Chr7:13362578..13367720	*CaDA2*	3640	1584	527	157–209	59.8009	5.20	-0.665	Nucl: 14
*CaLIM13*	ANPC01014064 (Ca7_1584)	LOC101489797	Chr7:37048198..37050170	*CaPLIM2b*	1581	633	210	9–61 & 103–155	23.2743	6.49	-0.426	Nucl: 8, Cyto: 5
*CaLIM14* *	ANPC01014952-53 (Ca8_146/147)	LOC101503031	Chr8:4482007..4487358	*CaDAR3*	4800	2178	725	406–458	83.1475	7.00	-0.538	Nucl: 7, Cyto: 5, Plas: 1
*CaLIM15*	ANPC01014955-56 (Ca8_149/150)	LOC101504663	Chr8:4654891..4660009	*CaDA3*	4185	1599	532	165–217	60.8637	5.94	-0.664	Nucl: 11, Mito: 2

*Predicted as Pseudo-gene in Bioproject: PRJNA175619

### Mapping on chromosomes and Genomic Organization of *LIM* genes

All the identified LIM domain coding genes (*CaLIMs*) were positioned on chickpea chromosomes. Except chromosome I, all others bear at least one *CaLIM*. The highest number of *CaLIMs* was observed on chromosome IV, VI and VII, each with 3 *CaLIMs* (Table 1). To identify gene structure, the ORF of each gene were analysed with BLASTN in publically available chickpea genome. Since *CaLIM14* is recognised ‘pseudo’ and no predicted mRNA is available, we have used the boundary demarcated in its gene (LOC101503031) as possible intron cleavage site. The exon region thus achieved after some manual editing revealed deduced protein sequence in single frame. The sequence analysis of all predicted LIM domain containing genes (15) revealed presence of introns (Fig 3B). The number of intron for each category such as 2LIMs and DA1/DAR proteins were consistently observed as 4 and 10 or 11, respectively (Fig 3B).

### Nomenclature and phylogenetic analysis of LIM proteins

To establish the relation and nomenclature, phylogenetic tree was constructed using MEGA6.0. The retrieved sequences for the protein sequences of *A*. *thaliana*, *B*. *rapa*, *O*. *sativa* and *C*. *arietinum* coding for LIM domain proteins were used (S2 Table). Phylogenetic tree was constructed using deduced LIM proteins with representation of both 2LIM and DA1/DAR members (Fig 1). This phylogeny clearly distributed the chickpea LIM domain proteins into two LIM domain containing proteins, henceforth called as Ca-2LIMs and Ca-DA1/Ca-DARs. The pair-wise amino acid sequences were also compared among all LIM proteins using BLAST analysis. The analysis indicated 20 to 91% identity among LIM domain containing proteins (S3 Table). More than 90% identity between CaLIM4 (CaWLIM1a) and CaLIM6 (CaWLIM1b) has demonstrated their close relation

Additional phylogenetic investigation was performed to substantiate position of two LIM domain containing proteins of chickpea and to rename as per the consensus established (Fig 2). The tree was constructed with other 2LIMs which comprised of 47 proteins with representation of *A*. *thaliana* (6), *O*. *sativa* (6), *C*. *arietinum* (9), *P*. *trichocarpa* (12) and *B*. *rapus* (14) (S2 Table). The position of most Ca-2LIMs was clear in this phylogeny, except CaLIM3 that needs further scrutiny (Fig 2). The CaLIM3 neither came adjacent to WLIM1 nor with GLIM1 (XLIM1) members. The limitation of this phylogeny was absence of PLIM1 members in included genus, which is also close to WLIM1 and GLIM1 proteins. PLIM1 is another important 2LIM protein frequent in *Solanaceae* and *Asteraceae* families [5]. To deal with this limitation, another phylogeny was constructed with exclusive members of PLIM1 categories (S3 Fig, S2 Table). We have also taken few Ca-2LIMs as out group members. This tree further suggested the resemblance of CaLIM3 with PtGLIM1a (PtXLIM1a) and PtGLIM1b (PtXLIM1b); hence we re-named it as CaGLIM1 (CaXLIM1).

The *CaLIM1*, *CaLIM5*, *CaLIM8*, *CaLIM12*, *CaLIM14* and *CaLIM15* appeared in common clad along with established DA1/DAR proteins (Fig 1, Table 1). DA1 and DA1-related (DAR) proteins are plant specific LIM proteins [27]. The common phylogeny indicated one clad specific to this protein sub-family that was distant from conventional 2LIM proteins (Fig 1). Structurally, DA1 comprised of two UIMs, single LIM-domain and conserved C-terminal amino acid sequences (DUF3633). The DAR (DA1-related) proteins is another variation and does not possess two UIMs, but otherwise similar to DA1. Thus, based on both phylogeny, chickpea LIM genes were re-named in order of their appearance in chromosomes as *CaDAR1*, *CaPLIM2a*, *CaGLIM1 (CaXLIM1)*, *CaWLIM1a*, *CaDA1*, *CaWLIM1b*, *CaβLIM1a*, *CaDAR2*, *CaWLIM2*, *CaδLIM2*, *CaβLIM1b*, *CaDA2*, *CaPLIM2b*, *CaDAR3* and *CaDA3* (Table 1). Since the DA1 members has been recently reported in Soybean [6]. We were equally interested to analyse the relation of Ca-DA1/DAR and DA1 members of cultivated soybean *G*. *max*. The phylogenetic tree for DA1/DAR proteins of *C*. *arietinum* and *G*. *max* clearly diverged into Class-I and Class-II proteins as earlier described [6]. This investigation suggested that most of the chickpea proteins have orthologs in *G*. *max*. Moreover, all such Gma-DA1 proteins have paralogous gene pairs, which was not the case of chickpea (S4 Fig). Interestingly, GmaDA1-1/GmaDA1-4 of *G*. *max* and CaDA3/CaDAR3 of *C*. *arietinum* do not have orthologous genes in other genus.

### Motif analysis of CaLIMs

All the deduced protein sequences of CaLIMs were subjected for motif analysis using MEME suite (Fig 3C). A total of 10 such motifs were identified and presented in S5 Fig. LIM domains are represented by motif 3, 2 and 1 in the same order; however, in case of DA1/DAR proteins the LIM domain is represented by motif 10, 2 and 1. The logo depiction for LIM domains of Ca-2LIM and DA1/DAR proteins were also given. In DA1/DAR proteins, amino acid sequences at C-terminal to LIM domain was found highly conserved with the consistent presence of motif 6, 5, 3, 8, 4 and 7 in the same order except CaDAR3 which lacks motif 7. Additionally, the N-terminal to LIM domain possesses motif 9 in CaDA1, CaDA2, CaDA3 and CaDAR3.

### *In silico* expression analysis of CaLIMs in different developmental stages

The search for “transcription factor family” for “LIM” in CTDB database identified 13 members (S4 Table). Among them, TC03126 and TC22392 as well as TC06070 and TC19129 were found similar. Therefore, larger identifier was selected for *in silico* expression studies. With our prediction using “CDC Frontier”, we were able to identify 15 LIM domain coding genes in chickpea genome (Table 1). In order to find the possibility of other LIMs the residual LIM genes (*CaPLIM2a*, *CaβLIM1b*, *CaDAR3* and *CaDA3*) were directly searched for BLASTN analysis in CTDB database using predicted gene sequences. The identifier TC08835 and TC33533 was found similar to *CaDA3*. Since, TC08835 was found 100% identical to *CaDA2*, we consider TC33533 as *CaDA3* (S4 Table). The close relation of both of them was also evident in phylogeny (Fig 1). The *CaDAR3* (LOC101503031) showed similarity with TC08390, which belongs to disease resistance protein (CC-NBS-LRR class) family. The BLAST search further revealed its closeness to different gene (LOC101505949), which is near to *CaDAR3* in genome. Since the deduced protein of TC08390 exhibited only RPW8 and NB-ARC, hence we have not considered it for *in silico* expression study. Other predicted *CaLIMs (CaPLIM2a* and *CaβLIM1b)* showed “no hit” in CTDB database.

Possible biological role of a gene can be easily visualized by its spatial or temporal expression pattern during development or stress-related cues. This helps in the foundation of future experiments for individual gene characterizations. The available transcriptome of chickpea (CTDB), provides a comprehensive expression data for various tissues representing both vegetative and reproductive features. Using the CTDB database [18,23–24], all the 12 *CaLIMs* expression data was retrieved and respective heat maps were generated (S6 Fig). In general, different stages of floral bud development and flowers do not show significant difference in both *Ca-2LIMs* and *DA1/DAR* sub-families. The LIM proteins similar to CRPs of animals (2LIMs) are very specific as far as their localization across different developmental tissues is concerned. Accordingly, they may have different functional relevance. We were also observed similar behaviour of *Ca-2LIMs* and with regard to different tissue type the variations are significant. The expression of *CaGLIM1*, *CaPLIM2b*, *CaβLIM1a* and *CaDA3* were not observed in vegetative tissue, however, their presence was noticed in samples of early growth phase and Flower bud stages (FB)/Flower stages (FL). The expression of *CaWLIM1a*, *CaWLIM1b*, *CaDA1*, *CaDA2* and *CaWLIM2* were mostly noticed in all tissues or growth stages, with reasonably high accumulation in vegetative tissues. The *CaδLIM2* was absent in most of vegetative tissue except mature leaves. However, its expression pattern reflects gradual increase in advance floral stage and remains consistent with different flower stages. The *CaDAR1* and *CaDAR2* were present in most of the tissues with more or less similar expression profile, except roots and mature leaves.

### Transcript expression analysis in different tissue representing multiple stages

**Fig 4:**
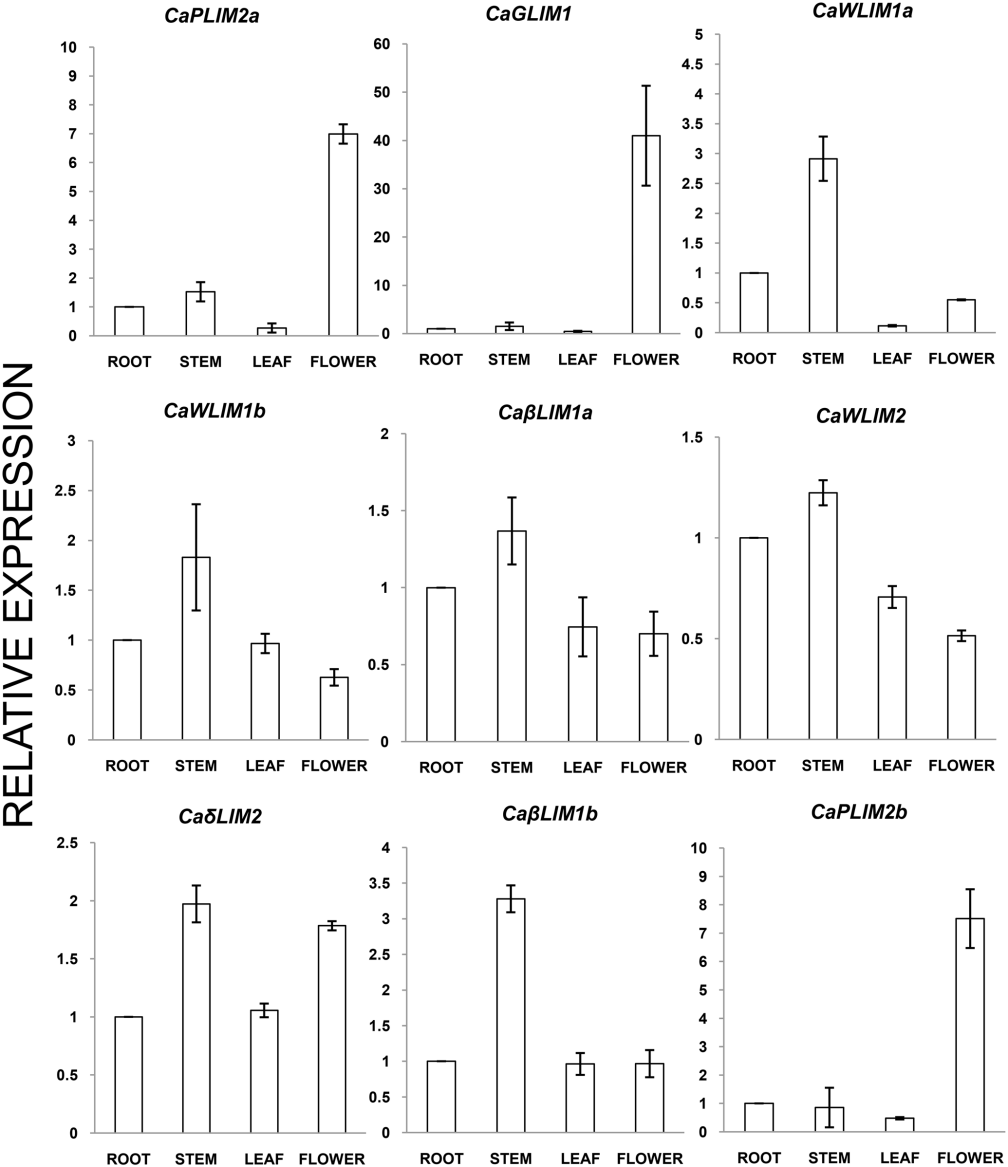
Expression profiles of *Ca-2LIM* genes across different developmental tissues. The expression data of *Ca-2LIM* genes in root, stem, leaf and flower were achieved through qRT-PCR. The expression values (Y-axis) were presented relative to root samples and EF-1α was taken as reference gene. Standard deviation of three replicates was indicated by error bars.

The two LIM domain containing proteins have been reported as key players in the regulation of actin dynamics and phenyl-propanoid pathway [8,9,13,15,28]. Accordingly, they expressed specifically in location related to these biological processes. The expression study was performed using specific primers (S1 Table) with cDNA of different tissue samples that have illustration of both vegetative and flowering growth stages i.e., roots, stem, leaves and flowers (Fig 4). The expression of *CaδLIM2*, *CaWLIM1a*, *CaWLIM1b*, *CaWLIM2*, *CaβLIM1a* and *CaβLIM1b* was found high in stem, followed by root, except *CaδLIM2*, where stem was followed by flower. Contrary to this, noteworthy higher transcription of *CaGLIM1*, *CaPLIM1a* and *CaPLIM1b* was observed in the floral tissues. Collectively, CTDB database and our own expression suggested absence of pseudo-gene in chickpea 2LIMs. All of them are transcriptionally active in one or more developmental conditions. This behaviour corroborates with the earlier findings of poplar *2LIMs*, where all the 12 *PtLIMs* were expressed in poplar tissues [29].

### Expression analysis under hormonal treatment

**Fig 5:**
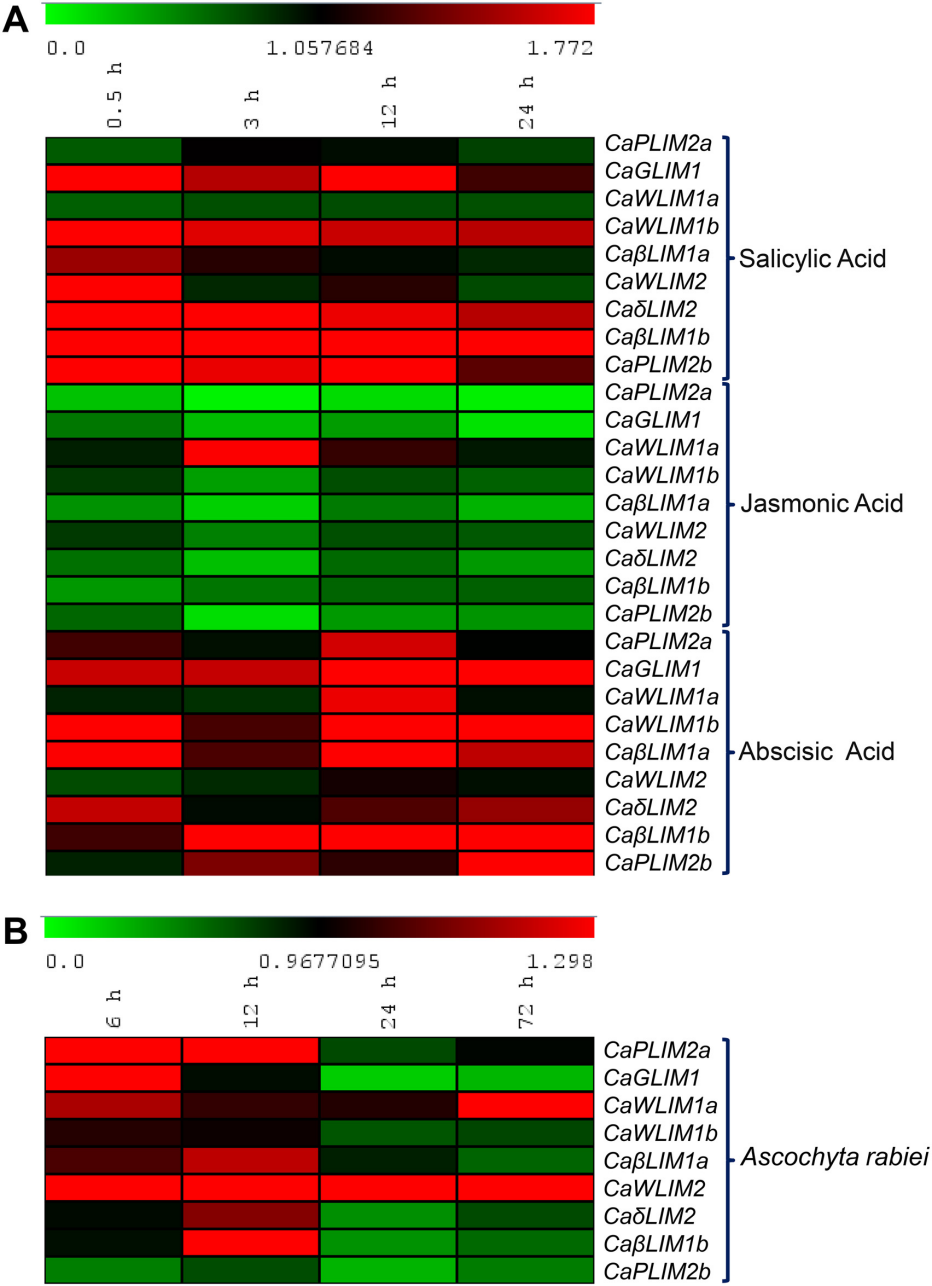
Heatmap representation of *Ca-2LIMs* expression in response to (A) SA, JA and ABA treatment (B) after spore inoculation of *Ascochyta rabiei*. Data were obtained through qRT-PCR for indicated time periods and presented relative to 0 h treatment. β-tubulin was taken as reference gene. Green and red color gradients indicate lower or higher transcript abundance, respectively.

In order to understand LIM expression in response to defense-related hormones, we investigated expression of *Ca-2LIMs* in plants treated with various hormones—*viz*. salicylic acid (SA), jasmonic acid (JA) and abscisic acid (ABA) (Fig 5A). The early (0.5 h) induction was observed in all *Ca-2LIMs* in response to SA, which falls at later stages (3 h onwards), with the exception of *CaWLIM1a* that showed immediate decline in transcript level. The expression showed increasing trend in *CaβLIM1b* and ~5 fold up-regulation was observed after 24 h of SA treatment. The expression of *CaWLIM2* and *CaGLIM1* showed biphasic expression pattern with higher transcript abundance at 0.5 h and 12 h. Contrary to SA treatment, JA mostly promotes down-regulation of *Ca-2LIMs*. Interestingly, higher expression of *CaWLIM1a* was observed in response to JA treatment. The trend of *CaWLIM2*, *CaGLIM1*, *CaδLIM2*, *CaWLIM1b* and *CaβLIM1a* showed consistent decline up to 3 h and found up-regulated thereafter. However, in case of *CaβLIM1b* initial decline in expression was observed followed by consistent increase. All the *Ca-2LIMs* exhibit up-regulation, in response to ABA, however, the expression of *CaWLIM2* was not much affected. The expression was remarkably high in *CaPLIM2b* and *CaβLIM1b*, where ~ 8 and ~ 3 fold up-regulation was observed, respectively.

### Expression analysis under *Ascochyta rabiei* infection

The investigation of *Ca-2LIM* genes was carried out in *A*. *rabiei* spore-inoculated plants after 0, 6, 12, 24 and 72 h (Fig 5B). The expression of *CaWLIM2*, *CaGLIM1*, *CaβLIM1a* and *CaPLIM2a* were found up-regulated immediately after *A*. *rabiei* spore inoculation, which remain high up to 12 h and decline thereafter. This drop in expression was comparatively more severe in *CaGLIM1* and *CaβLIM1a*; however, the *CaWLIM2* expression remains up-regulated. The expression of *CaδLIM2* and *CaβLIM1b* was found down-regulated, except for 12 h treated samples. The expression of WLIM1 members revealed disparity with more or less unaffected expression of *CaWLIM1a* and down-regulation of *CaWLIM1b*. The expression pattern of *CaPLIM2b* was noticed down-regulated.

## Discussion

**Fig 6:**
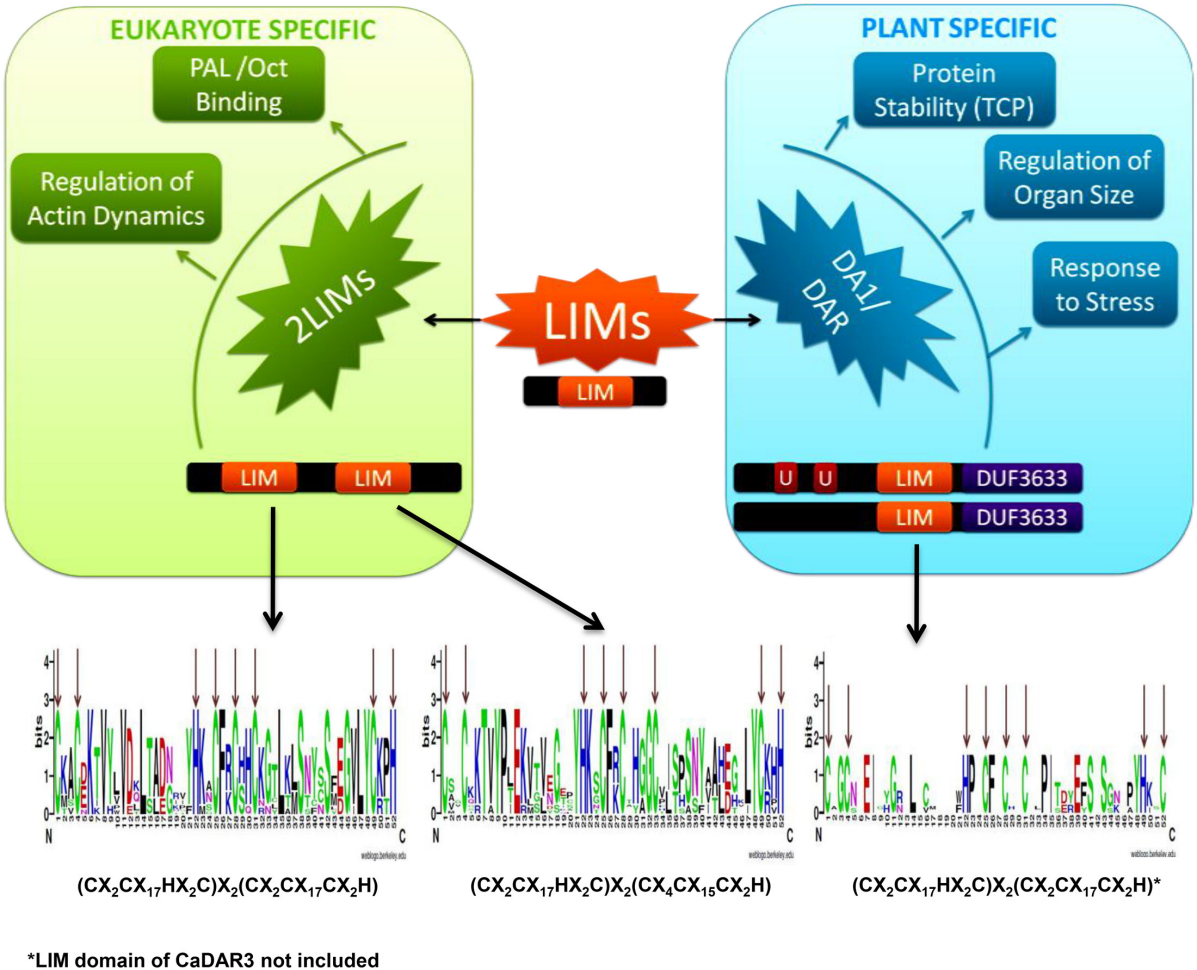
Functional relevance of LIM domain containing proteins in plants and structure of LIM domain in CaLIMs.

Plant being sessile, needs a precise mechanism to sustain their life and related developmental events. This led to the evolution of novel gene families or genes with new functions or structural diversification to established families. In context to distribution and divergence of gene families in eukaryotes, they may be exclusive to eukaryote lineage or specific to plant or animals. One of such eukaryotic lineage specific gene family is “LIM”, known for its versatility to influence various biological functions such as regulation of gene expression, cell adhesion and signal transduction [30]. Additionally, it has role in oncogenesis and also possesses protein-protein interaction motifs for such function [31]. The fascination for plant LIM family exists for last two decades, but limited evidence towards in depth analysis throughout the genome. Few report either targeting single type of LIM proteins or with other taking a number of plants has added new dimensions [5,6]. Recently the genome wide survey of *B*. *rapa* LIMs, has given a comprehensive account [14]. This protein family is known for regulation of actin dynamics, organ size, protein stability, stress response and metabolism in plants (Fig 6). In present study, chickpea as model/representative legume was chosen to investigate this important gene family in recently sequenced genome.

The present study exhibited presence of 15 LIM candidates in *C*. *arietinum*. The early divergence was observed which leads to separation of LIM proteins into two LIM domain proteins (Ca-2LIMs) and DA1/DAR proteins (Figs 1 and 3A). This separation is more visible in CLUSTALX2 and SMART analysis (S1 and S2 Figs), which revealed additional domains in DA1/DAR members. Nine members were found similar to cysteine-rich proteins of animals grouped as Ca-2LIMs. The architecture of plant two LIM domain containing proteins is different from animal CRPs. Here, the first LIM domain is similar to animal LIM [4], but the second LIM domain is represented by C-X_4_-C-X_15_-C-X_2_-H instead of C-X_2_-CX_17_-C-X_2_-H [32] (Fig 6). Compared with earlier investigated plant systems total number of such genes were found midway [5,14,29]. Six 2LIM proteins were observed in *Arabidopsis* and *Oryza* [5]. However, this number doubled to 12 in *P*. *trichocarpa* where duplication of almost every gene was noticed [29]. Recent genome wide study in *B*. *rapa* reflected 14 such candidates, with exception of *BrLIM6 (BrWLIM2b)* possessing single LIM domain [14]. The close look on high number of these LIMs, exhibited its strong association to either tree habit or genome multiplication. Since LIMs are known to provide influence over lignin biosynthesis, the higher number may easily presume to offer high lignin content or its strictly controlled biosynthesis. This is also evident in *Arabidopsis*, where such function is not presented by LIM proteins. The herbaceous habit and short life span of *Arabidopsis* may be the reason for low accumulation of lignified tissues. Moreover, *Populus* and *Brassica* LIM proteins revealed close paralogy in phylogenetic investigation. Similarly, among *Ca-2LIMs*, *CaPLIM2* and *CaβLIM1* showed possible paralogy with >90% bootstrap. Besides, both the members of *CaWLIM1* also showed closeness with low bootstrap support (Figs 1 and 2).

The Ca-2LIMs can be easily grouped into LIM1 and LIM2 members, which was also noticed in previous report [33]. These members were further classified into αLIM, βLIM, γLIM and δLIM. Moreover, the presence of WLIM1, WLIM2 and PLIM2 members are also visible in Ca-2LIMs (Fig 2). The Ca-2LIMs were found in corroboration with proposed classification [5,14] and re-named accordingly (Table 1). The close homology with established PLIM1 members suggested absence of PLIM1 candidate in chickpea, which is also a feature of *Arabidopsis*, *Brassica*, *Populus* and *Oryza* (Fig 2, S3 Fig). Moreover, the strong relationship between phylogenetic classification and expression across developmental stages of Ca-2LIMs were observed (Figs 2 and 4). The expression of *CaPLIMs* and *CaGLIM1* were found higher in flowers indicating expression in floral tissues like pollen, as reported earlier. However, CaGLIM1 occupy same clade with PtGLIM1a and PtGLIM1b in phylogeny, which is known for high expression in secondary xylem [29]. Although, CaGLIM1 structurally similar to PtGLIMs, but it follows expression pattern similar to the PLIM1 members. The expression profile of this category of LIM indicated their role in the regulation of events related to reproduction and pollen development [12,34]. Thus, expression pattern of *CaGLIM1* further supported the possibility of neofunctionalization as speculated for PtLIMs [29,35]. HaPLIM1 the first identified LIM, was found to play role in pollen germination and growth of pollen tube [11]. Preferential expression of *AtPLIM2a*, *AtPLIM2b* and *AtPLIM2c* were reported in pollen grains [12]. The same group has also reported the inhibitory effect of high pH and Ca^++^ concentration on activity of PLIM2 members. Contrast to this, other LIMs (*AtWLIM* members) was found ubiquitously with pH and Ca^++^independent activity. Similar role for *GhPLIM1* was also suggested anther-specific expression and regulation of actin cytoskeleton [34].

The expression of other *Ca-2LIMs* revealed ubiquitous nature with higher accumulation in stem tissues. This corroborates with the expression pattern for most of the WLIMs studied till now [12]. The higher expression of these LIMs was observed in tissue which has role particularly in providing strength to the plant. The land plants acquired their erect texture and height due to fortification of tissues, particularly stem. This strength comes due to the deposition of lignin which is one of the characteristic differences between land plants and non-land plant forms. Therefore, higher expression of these LIMs indicated potential role of few Ca-2LIMs in the regulation of lignin biosynthesis by modulating phenyl-propanoid pathway. This pattern of regulation of lignin biosynthesis by 2LIM proteins were also observed earlier in *Nicotiana*, *Eucalyptus* and *Gossypium* [8,13,15,36]. Additionally, the enhanced stability of actin cytoskeleton by NtWLIM1 was reported [37]. The WLIM1-GFP was expressed in BY2 cells and showed delayed depolymerisation of actin cytoskeleton induced by Latraculin B. The NtWLIM1 over-expression in *N*. *benthamiana* leaves showed fewer and thicker actin bundles. The individual LIM domain can directly interact with actin filaments; however, deletion of any domain reduces F-acting binding and bundling [9]. Similar function was also observed for other WLIM members such as AtWLIMs and GhWLIM5 [12,38]. The significance of WLIM members in the transcription regulation of histone biosynthesis was also demonstrated [16]. Interestingly, NtWLIM2 and GhWLIM1a were recently recognised to perform more than one function [13,16].

The DA1/DAR of LIM family proteins are plant specific, which offer regulation of organ size and plant defense response [10,39–41]. The gene structure of these proteins comprised of single LIM domain and highly conserved C-terminal ends. The N-terminal contains a number of diverse domains such as UIM, TIR-NB-LRR which offers structural and functional diversity. Among six DA1/DARs, only three (CaDA1, CaDA2 & CaDA3) possess 2 UIM domains (S1 Fig). The availability and mostly universal expression pattern by *in silico* expression data analysis revealed the absence of pseudogene except CaDAR3. The expression of *CaDA1* and *CaDA2* was observed high in vegetative tissues. Contrast to this, *CaDA3* was found mostly in floral tissues and absent in vegetative tissues. Moreover, *CaDAR1* and *CaDAR2* was absent in roots and mature leaves. Interestingly, the consistent presence across floral tissue/stages is largely in accordance with *BrDARs* [14]. The functional relevance of this LIM sub-family was studied in *Arabidopsis* and more recenty in *Glycine* [7,10,39–43]. Our study also suggested most of the Ca-DA1/DAR proteins have their orthologous candidate protein in *G*. *max* and found further diversifed in later. The most common form in *Arabidopsis* has two UIM at N-terminal which interacts with ubiquitin. These forms regulate size of various organs in context dependent manner. Another variant of DA1 is TIR-NB-LRR-LIM protein (AT5G17890), which is regulated in temperature dependent manner and modulates growth, cell death and freezing tolerance [39]. However, this form of LIM protein was absent in chickpea or other plant except *Camelina sativa*. Addition to this, the recent studies of *Glycine* DA1 members of cultivated and wild variety suggested exceptionally high identities between orthologous gene pairs. But their expression pattern varies in response to abiotic stress. Interestingly, the overexpression of *GsoDA1* improved salt tolerance in transgenic *Arabidopsis* and no alteration in seed size was observed [43].

To understand the significance of *Ca-2LIMs* during stress, we investigated their response to defense hormone (SA, JA and ABA) and, *A*. *rabiei* a necrotrophic pathogen of chickpea (Fig 5). The plant hormones are key players of plant responses and known for direct impact on plant survival. Many stresses influence their production, which in turn channel plant responses. Furthermore, LIM genes are not much explored against biotic stresses except *B*. *rapa* and *F*. *oxysporium* interactions [14]. The *A*. *rabiei* has been considered recently as a model necrotrophic fungus [44–46] which was used in this study. Largely, *Ca-2LIMs* was found modulated in comparison with control. Altogether, the treatment of SA, ABA and *A*. *rabiei* was found up-regulated for most of the genes. In contrast, JA leads to down-regulation except CaWLIM1a. The modulation of *Ca-2LIMs* in response to exogenous supply of key players of plant defense such as SA and JA [47] advocated their relevance in plant immune response. To our knowledge, LIM genes are not investigated so far in response to SA and JA treatment, though their modulation against ABA, *F*. *oxysporium*, cold and pH stress were recently reported [14]. The regulation of *Ca-2LIMs* in response to ABA corroborates with the expression pattern of *BrLIMs*. Furthermore, high expression of *CaWLIM2* in response to *A*. *rabiei* also supported by *F*. *oxysporium* where high expression was observed in a *Brassica* ortholog *BrWLIM2c* [14].

## Conclusion

In nut shell, the genome-wide identification, efficient classification and expression analyses of *C*. *arietinum* LIM genes offer an insight on their potential contribution in growth, development and stress-related processes. The deduced protein analysis demonstrated two distinct sub-families differing in functional domains. These were designated as Ca-2LIMs and Ca-DA1/DAR, based on their resemblance to animal CRPs and plant-specific DA1, respectively. The specific expression pattern of *Ca-2LIMs* revealed their significance in the regulation of crucial events related to development. Moreover, their regulation under hormone (SA, JA and ABA)-treated and *A*. *rabiei* infected samples offers considerable support to visualize and explore their function in relation to biotic stress. Over all, investigation of this gene family in economically important legume crop will open new possibility on crop development and further study of various biological phenomenon or functions.

## Supporting Information

S1 Fig Depiction of various domains in *Cicer arietinum* LIM proteins using SMART analysis. (A) Ca-2LIMs (B) Ca-DA1/DAR.
(PDF)

S2 Fig Alignment of deduced amino acid sequences of CaLIMs and other homologous proteins. (A) 2LIM and (B) DA1/DAR protein groups using CLUSTALX2. Boxes indicate UIM (Green), LIM (Pink) and Conserved C-terminal (Blue) domains. The conserved cysteine and histidine of LIM domain is marked by astrix.
(PDF)

S3 Fig Phylogenetic analysis of CaLIM3 (CaGLIM1) along with other PLIM members. (PDF)

S4 Fig Phylogenetic analysis of Ca-DA1/DAR and GmaDa1 proteins. The Maximum likelihood tree was constructed using protein sequences. Blue closed circles were used to show Ca-DA1/DAR proteins. Two major classes were presented in different colours.
(PDF)

S5 Fig Conserved motifs identified in CaLIMs through MEME analysis. (PDF)

S6 Fig Heatmap representation for *in silico* expression of *CaLIM* genes in different tissues as retrieved from CTDB. (A) Expression in shoots, roots, mature leaves, flower buds and young pods, generated by 454 pyrosequencing of cDNA libraries prepared from respective samples. (B) Expression in germinating seedling (GS), young leaf (YL), shoot apical meristem (SAM), flower bud stages (FB1-FB4) and flower stages (FL1-FL4) generated by Illumina sequencing of RNA-seq libraries prepared from respective samples. Green and red color gradients indicate lower or higher transcript abundance, respectively.
(PDF)

S1 Table Primer of *Ca-2LIM* genes used for Real Time-PCR analysis. (PDF)

S2 Table Deduced protein sequences used for phylogenetic investigation. (XLS)

S3 Table Pair-wise amino acid sequence comparisons illustrated as percent identity among members of the CaLIM proteins. (PDF)

S4 Table Identifier retrieved from CTDB for *in silico* expression analysis. (PDF)
