# Correction: Genome Wide Identification of *LIM* Genes in *Cicer arietinum* and Response of *Ca-2LIMs* in Development, Hormone and Pathogenic Stress

**DOI:** 10.1371/journal.pone.0141900

**Published:** 2015-10-27

**Authors:** Vikas Srivastava, Praveen Kumar Verma

There is an error in the seventh sentence of the “Plant materials, tissue collection, hormone treatment and A. rabiei infection” section of the Material and Methods. The sentence should read: The spore suspension of freshly grown *A*. *rabiei* cultures (2 X 10^5^ spores/mL) were sprayed onto 3 week old phytotron-grown plants and almost one-third of the aerial part of the respective samples were collected in triplicate after 0, 6, 12, 24 and 72 h.

There is an error in the ninth sentence of the fourth paragraph of the Discussion. The sentence should read: The WLIM1-GFP was expressed in BY2 cells and showed delayed depolymerisation of actin cytoskeleton induced by Latrunculin B.
