# Supplementary material for: Genome Wide Identification of LIM Genes in Cicer arietinum and Response of Ca-2LIMs in Development, Hormone and Pathogenic Stress
Source: PLoS One. 2015 Sep 29;10(9):e0138719. doi: 10.1371/journal.pone.0138719 (PMC4587737; doi:10.1371/journal.pone.0138719)

S1 Fig. Depiction of various domains in *Cicer arietinum* LIM proteins using SMART analysis. (A) Ca-2LIMs (B) Ca-DA1/DAR

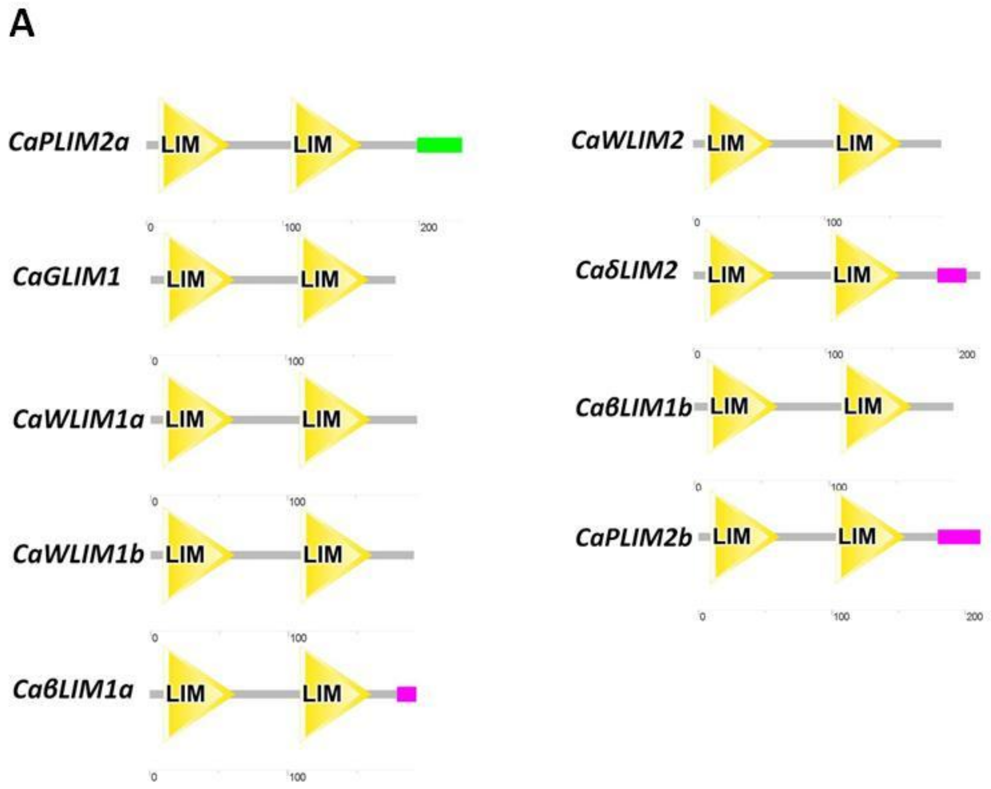

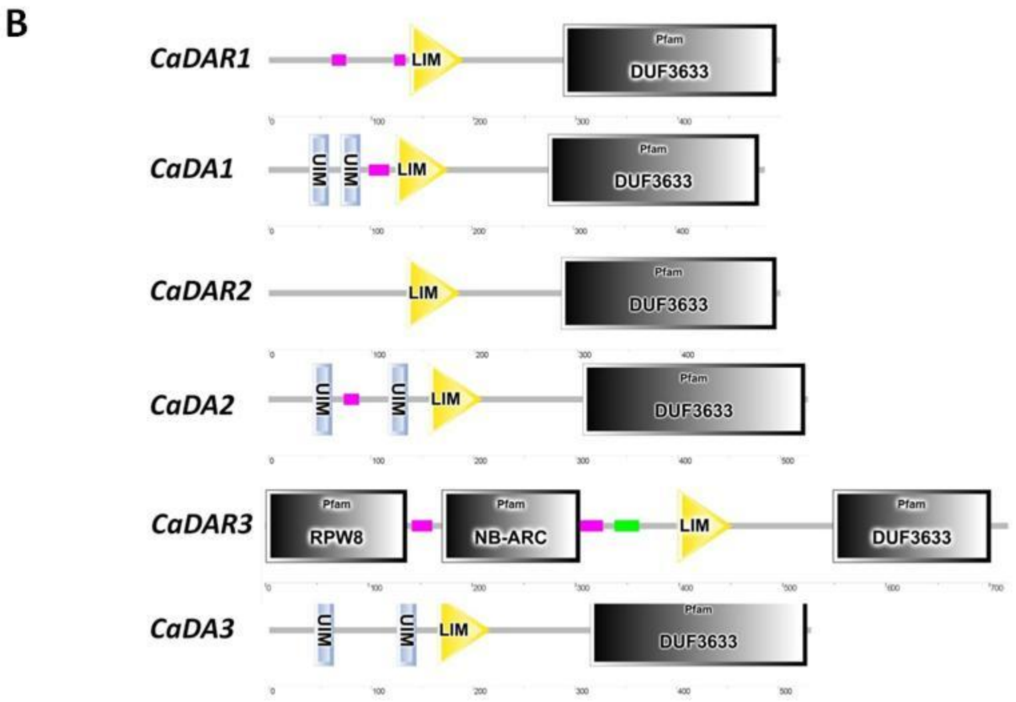

Supplement: S1 Fig — (A) Ca-2LIMs (B) Ca-DA1/DAR. (PDF) [file pone.0138719.s001.pdf]
