# Supplementary material for: Genome Wide Identification of LIM Genes in Cicer arietinum and Response of Ca-2LIMs in Development, Hormone and Pathogenic Stress
Source: PLoS One. 2015 Sep 29;10(9):e0138719. doi: 10.1371/journal.pone.0138719 (PMC4587737; doi:10.1371/journal.pone.0138719)

S2 Fig. Alignment of deduced amino acid sequences of CaLIMs and other homologous proteins. (A) 2LIM and (B) DA1/DAR protein groups using CLUSTALX2. Boxes indicate UIM (Green), LIM (Pink) and Conserved C-terminal (Blue) domains. The conserved cysteine and histidine of LIM domain is marked by astrix.

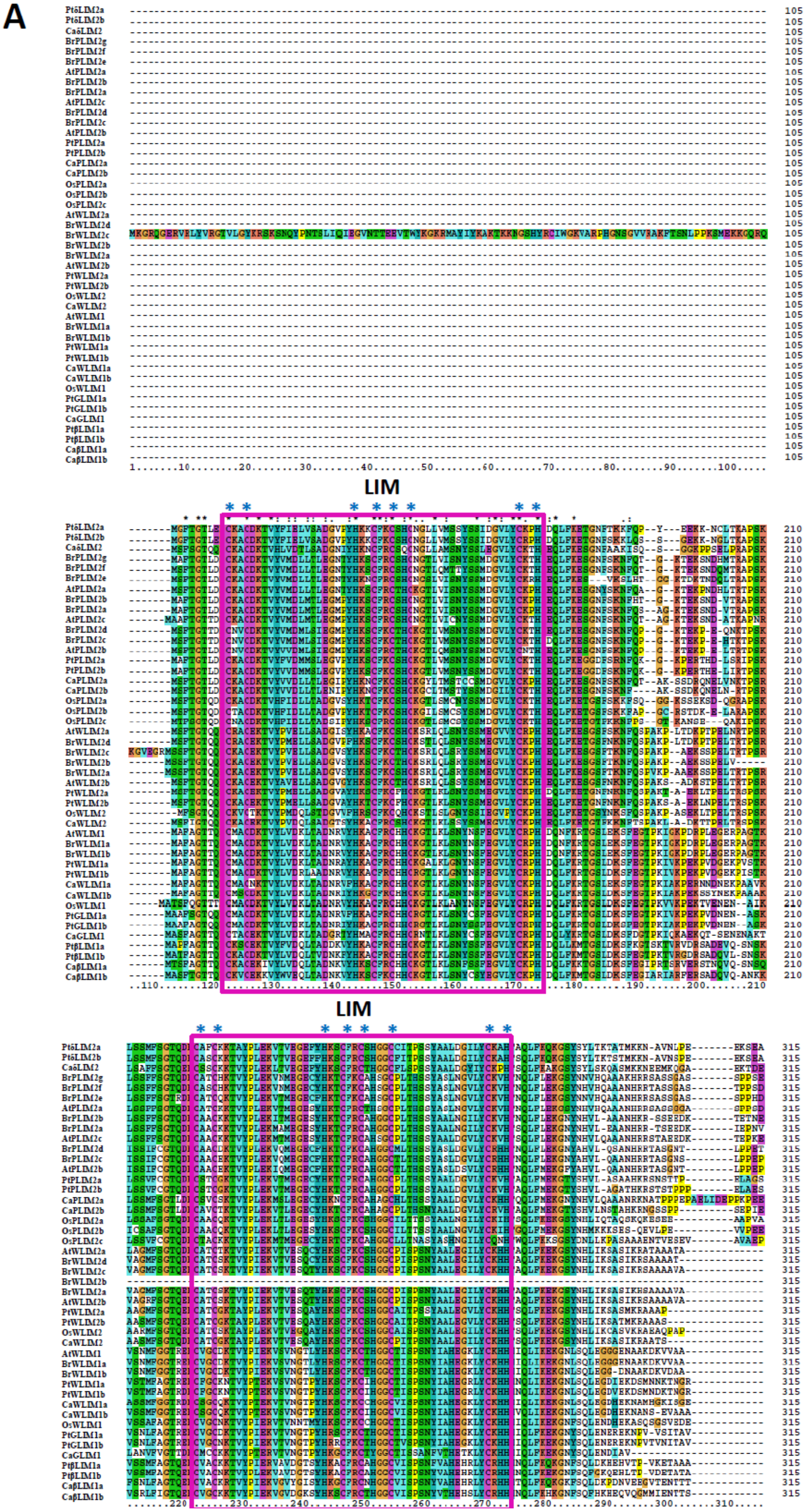

B

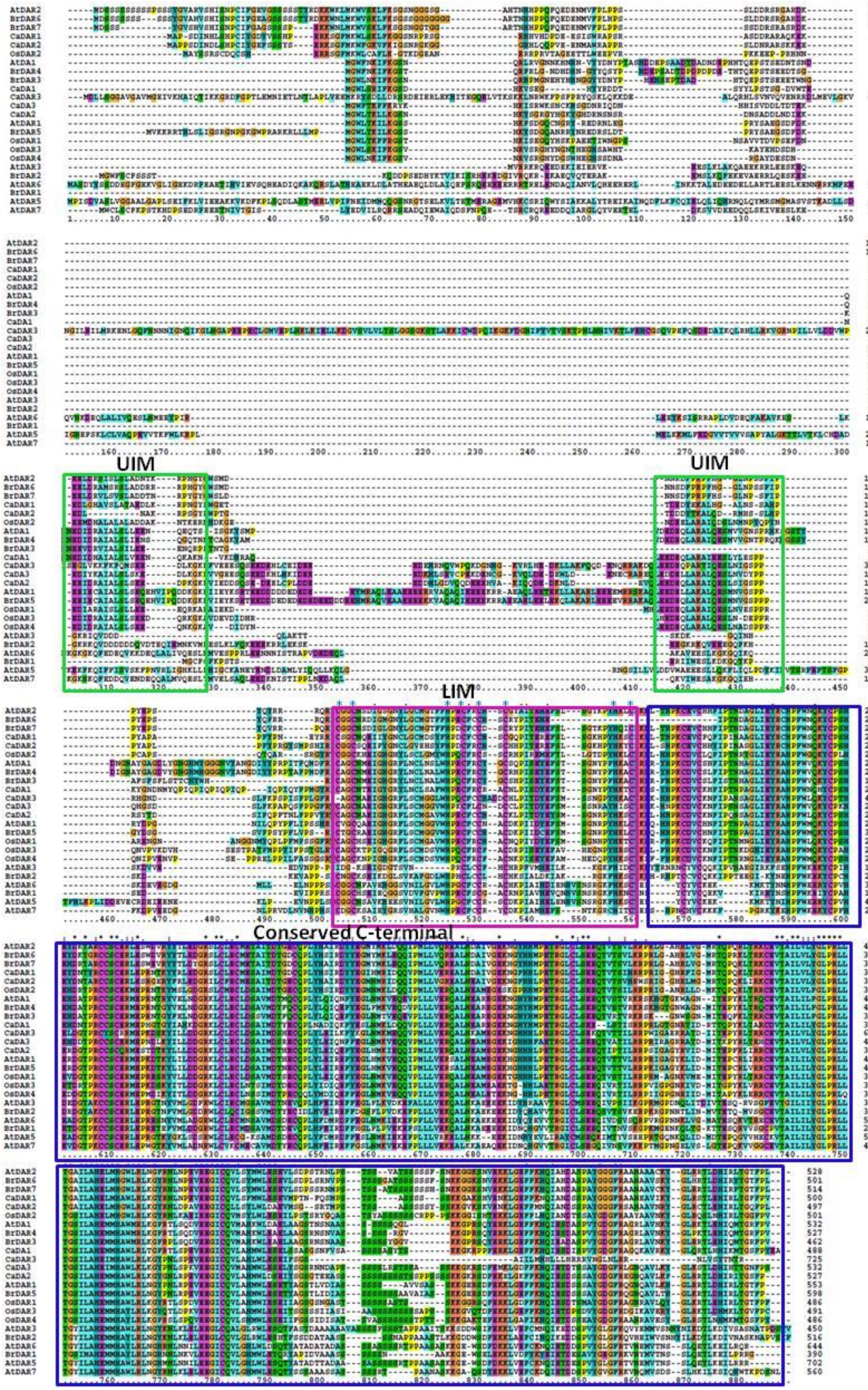

Supplement: S2 Fig — (A) 2LIM and (B) DA1/DAR protein groups using CLUSTALX2. Boxes indicate UIM (Green), LIM (Pink) and Conserved C-terminal (Blue) domains. The conserved cysteine and histidine of LIM domain is marked by astrix. (PDF) [file pone.0138719.s002.pdf]
