# Supplementary figures and images for: Genome Wide Identification of LIM Genes in Cicer arietinum and Response of Ca-2LIMs in Development, Hormone and Pathogenic Stress
Source: PLoS One. 2015 Sep 29;10(9):e0138719. doi: 10.1371/journal.pone.0138719 (PMC4587737; doi:10.1371/journal.pone.0138719)

**S3 Fig.** Phylogenetic analysis of CaLIM3 (CaGLIM1) along with other PLIM members.

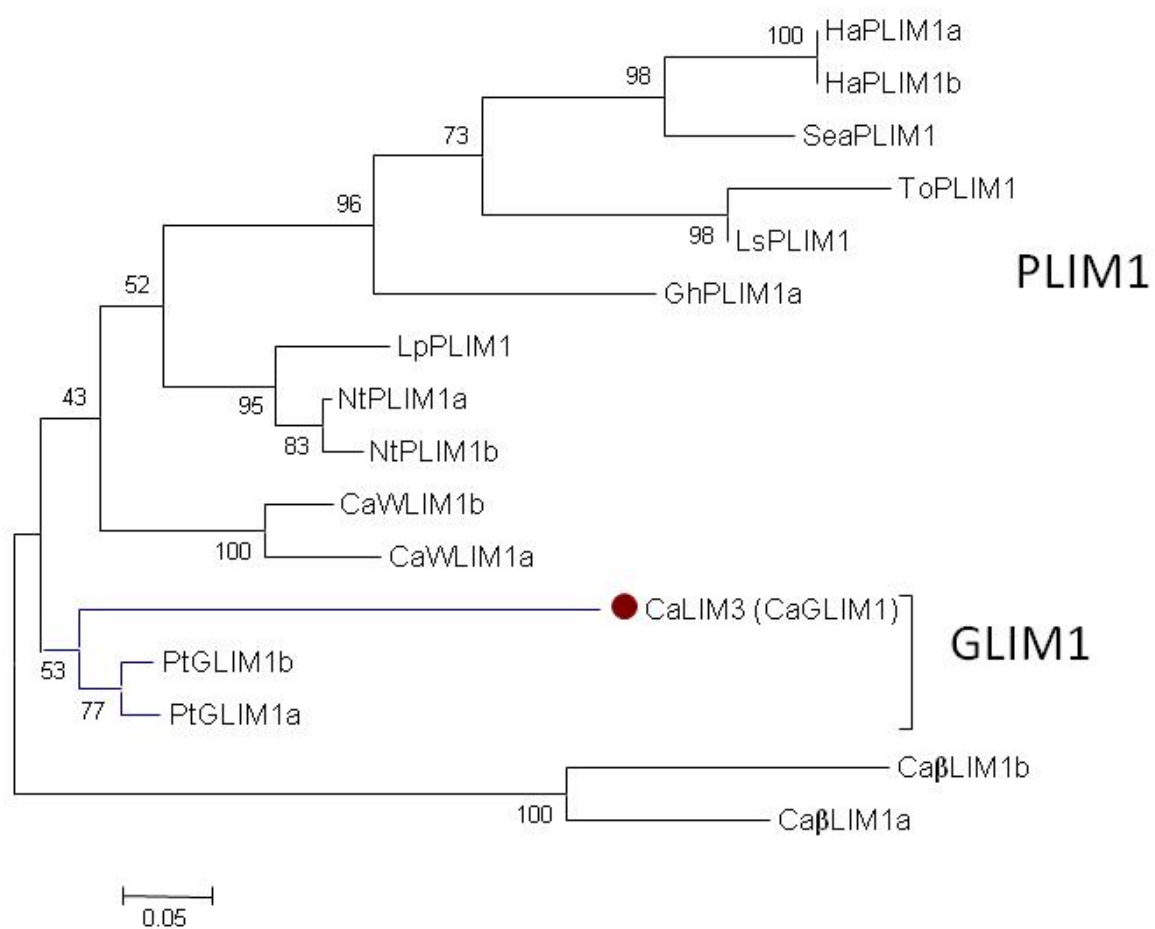

Supplement: S3 Fig — (PDF) [file pone.0138719.s003.pdf]

S5 Fig. Conserved motifs identified in CaLIMs through MEME analysis.

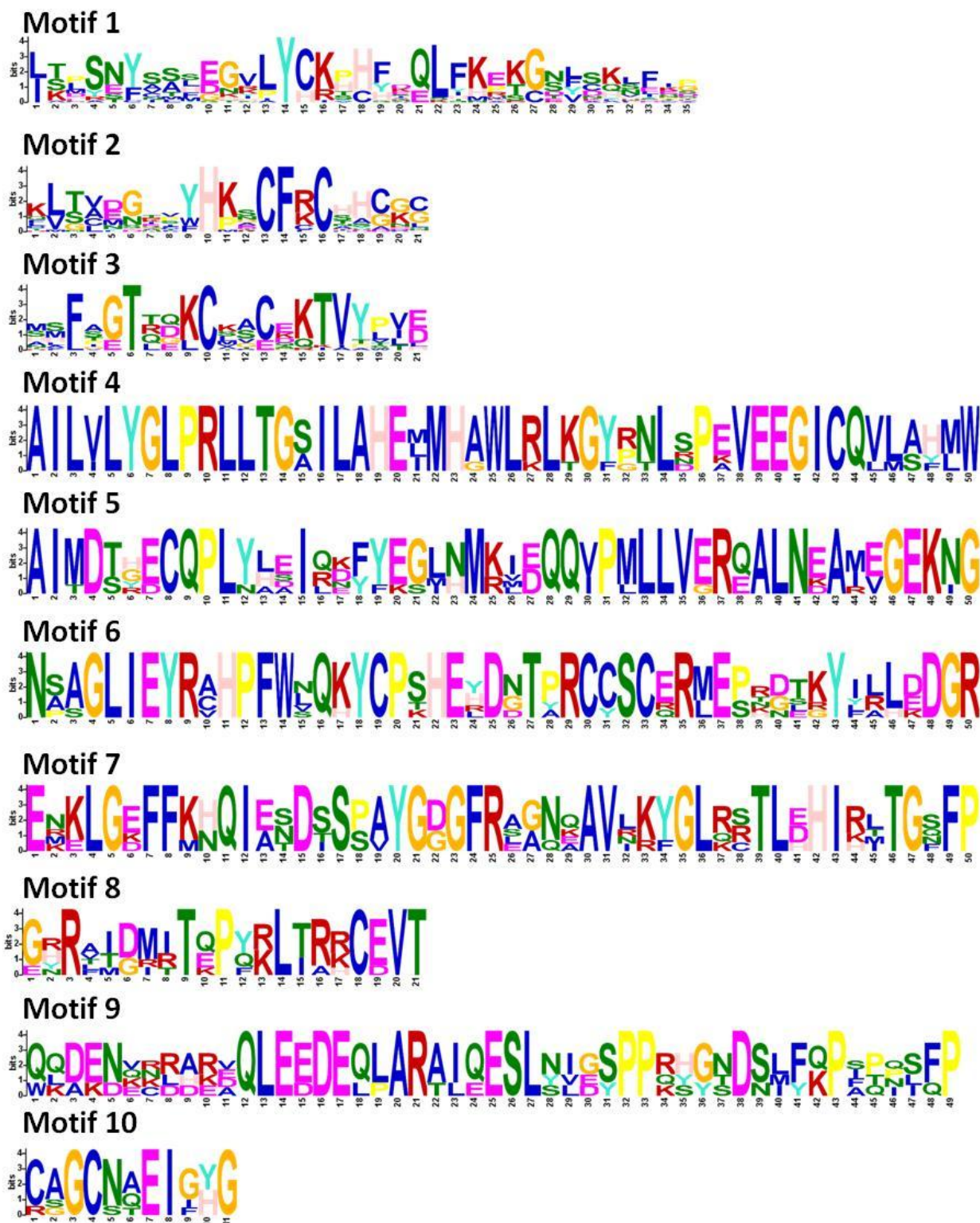

Supplement: S5 Fig — (PDF) [file pone.0138719.s005.pdf]
