# Supplementary material for: Genome Wide Identification of LIM Genes in Cicer arietinum and Response of Ca-2LIMs in Development, Hormone and Pathogenic Stress
Source: PLoS One. 2015 Sep 29;10(9):e0138719. doi: 10.1371/journal.pone.0138719 (PMC4587737; doi:10.1371/journal.pone.0138719)

S4 Fig. Phylogenetic analysis of Ca-DA1/DAR and GmaDa1 proteins. The Maximum likelihood tree was constructed using protein sequences. Blue closed circles were used to show Ca-DA1/DAR proteins. Two major classes were presented in different colours.

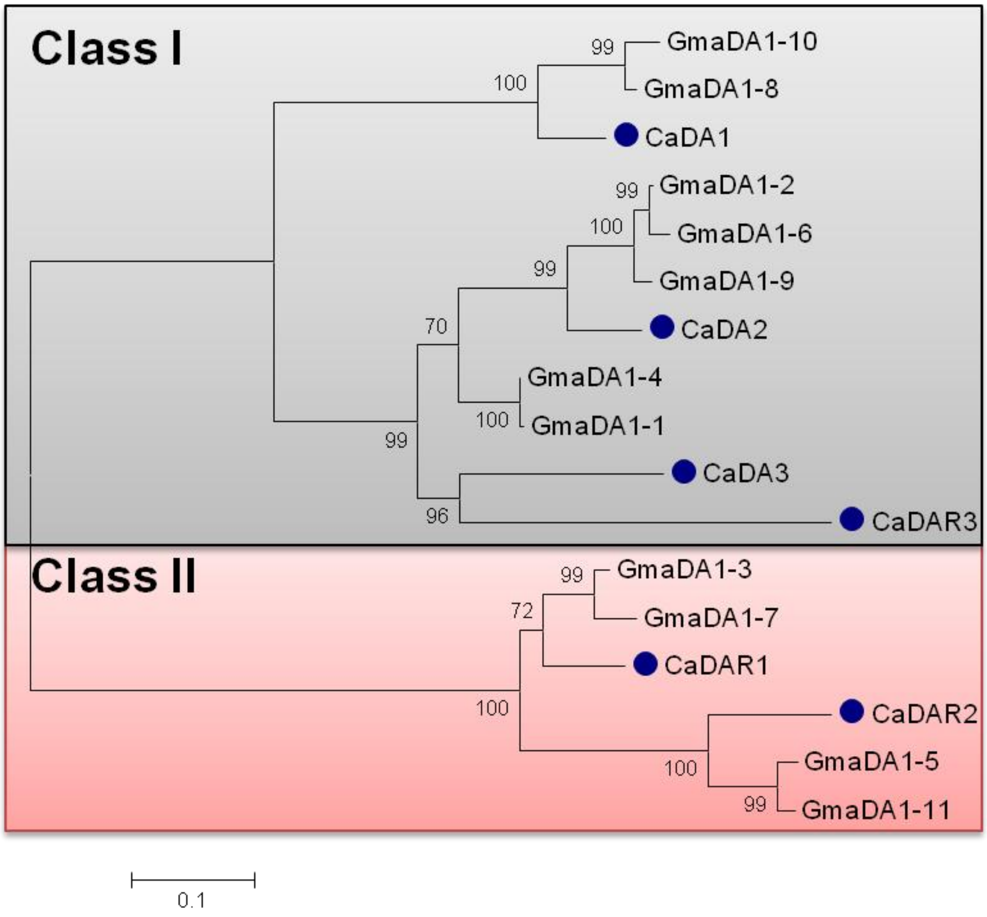

Supplement: S4 Fig — The Maximum likelihood tree was constructed using protein sequences. Blue closed circles were used to show Ca-DA1/DAR proteins. Two major classes were presented in different colours. (PDF) [file pone.0138719.s004.pdf]
