# Supplementary material for: Genome Wide Identification of LIM Genes in Cicer arietinum and Response of Ca-2LIMs in Development, Hormone and Pathogenic Stress
Source: PLoS One. 2015 Sep 29;10(9):e0138719. doi: 10.1371/journal.pone.0138719 (PMC4587737; doi:10.1371/journal.pone.0138719)
Supplement: S1 Table — (PDF) [file pone.0138719.s007.pdf]

S1 Table. Primer of *Ca-2LIM* genes used for Real Time -PCR analysis

Gene		Forward (5'-3')	Reverse (5'-3')
CaLIM2	*CaPLIM2a*	TCATGGAGAAGGGAAATTACAATCA	GGAGGTGGAGTTGCATTCTTTCT
CaLIM3	*CaGLIM1*	TGAGACAAAGCTCTACTGCAAACA	TCGAGCTGACTGTAATTTCCTTTCT
CaLIM4	*CaWLIM1a*	AGGGAAATTTAAGCCAACTAGAAGGT	CGCTGATTTTCCCATGCAT
CaLIM6	*CaWLIM1b*	AGCACACGAAGGAAAACTGTATTG	GGTCACCTTCAAGCTGGCTTA
CaLIM7	*CaβLIM1a*	CCACCATACTCAATTGTTCAAGCA	TCAGTCACCCCTTCTTCTACATTGT
CaLIM9	*CaWLIM2*	TCAATCAAGCGTGCAGCAA	CAAAGGCAATAGAAGGCACCAAAC
CaLIM10	*CaδLIM2*	CAGCAGCAACATCAGACACAGA	CTTGTTGAGAGCCTAATCCTCTTGT
CaLIM11	*CaβLIM1b*	GACACCACCATAATCAACTCTTCAA	TGCTCATGCTTGTGAAATTGG
CaLIM13	*CaPLIM2b*	CAAGGAATCTGGCAATTTTAGCA	CTTGGTGTCCTATTCAGCTCATTTT
CaEF1a	-	TCCACCACTTGGTCGTTTTG	CTTAATGACACCGACAGCAACAG
CaBTub	-	ATTCTGGTTAGGAGGCTGATGTG	TTAACCTCCGTTGCCCAAAT
