# Supplementary material for: Genome Wide Identification of LIM Genes in Cicer arietinum and Response of Ca-2LIMs in Development, Hormone and Pathogenic Stress
Source: PLoS One. 2015 Sep 29;10(9):e0138719. doi: 10.1371/journal.pone.0138719 (PMC4587737; doi:10.1371/journal.pone.0138719)
Supplement: S3 Table — (PDF) [file pone.0138719.s009.pdf]

S3 Table. Pair-wise amino acid sequence comparisons illustrated as percent identity among members of the CaLIM proteins.

	**CaPLIM2a**	**CaGLIM1**	**CaWLIM1a**	**CaWLIM1b**	**CaβLIM1a**	**CaWLIM2**	**CaδLIM2**	**CaβLIM1b**	**CaPLIM2b**	**CaDAR1**	**CaDA1**	**CaDAR2**	**CaDA2**	**CaDAR3**	**CaDA3**
**CaPLIM2a**	100														
**CaGLIM1**	46	100													
**CaWLIM1a**	51	72	100												
**CaWLIM1b**	53	71	91	100											
**CaβLIM1a**	49	63	68	68	100										
**CaWLIM2**	60	56	62	59	56	100									
**CaδLIM2**	51	49	55	53	52	64	100								
**CaβLIM1b**	50	62	66	69	77	55	51	100							
**CaPLIM2b**	76	46	52	49	51	63	61	52	100						
**CaDAR1**	25	50	35	41	27	35	20	29	25	100					
**CaDA1**	31	35	33	32	32	34	30	36	37	53	100				
**CaDAR2**	32	26	35	32	27	29	24	26	27	74	49	100			
**CaDA2**	31	28	32	37	25	32	28	30	24	56	58	59	100		
**CaDAR3**	37	31	22	53	28	35	33	33	47	66	64	51	72	100	
**CaDA3**	27	24	27	26	28	34	29	27	24	59	55	52	69	76	100
