# Supplementary material for: Genome Wide Identification of LIM Genes in Cicer arietinum and Response of Ca-2LIMs in Development, Hormone and Pathogenic Stress
Source: PLoS One. 2015 Sep 29;10(9):e0138719. doi: 10.1371/journal.pone.0138719 (PMC4587737; doi:10.1371/journal.pone.0138719)
Supplement: S4 Table — (PDF) [file pone.0138719.s010.pdf]

S4 Table. Identifier retrieved from CTDB for *in silico* expression analysis.

**Identifier**	**Contigs Assigned**	**Name Given**	**New Name**
TC03126/TC22392	Ca6_71/72	*CaLIM8*	*CaDAR2*
TC03418	Ca6_74	*CaLIM9*	*CaWLIM2*
TC06070/TC19129	Ca5_1730	*CaLIM6*	*CaWLIM1b*
TC06172	Ca4_2033	*CaLIM5*	*CaDA1*
TC08758	Ca4_473	*CaLIM3*	*CaGLIM1*
TC08835	Ca7_479	*CaLIM12*	*CaDA2*
TC13769	Ca6_174	*CaLIM10*	*CaδLIM2*
TC14248	Ca2_1375	*CaLIM1*	*CaDAR1*
TC18961	Ca4_1985	*CaLIM3*	*CaWLIM1a*
TC19748	Ca7_1584	*CaLIM13*	*CaPLIM2b*
TC32703	Ca5_1861	*CaLIM7*	*CaβLIM1a*
TC33533	Ca8_149/150	*CaLIM15*	*CaDA3*
