# Supplementary material for: Genome Wide Identification of LIM Genes in Cicer arietinum and Response of Ca-2LIMs in Development, Hormone and Pathogenic Stress
Source: PLoS One. 2015 Sep 29;10(9):e0138719. doi: 10.1371/journal.pone.0138719 (PMC4587737; doi:10.1371/journal.pone.0138719)

S6 Fig. Heatmap representation for *in silico* expression of *CaLIM* genes in different tissues as retrieved from CTDB. (A) Expression in shoots, roots, mature leaves, flower buds and young pods, generated by 454 pyrosequencing of cDNA libraries prepared from respective samples. (B) Expression in germinating seedling (GS), young leaf (YL), shoot apical meristem (SAM), flower bud stages (FB1-FB4) and flower stages (FL1-FL4) generated by Illumina sequencing of RNA-seq libraries prepared from respective samples. Green and red color gradients indicate lower or higher transcript abundance, respectively.

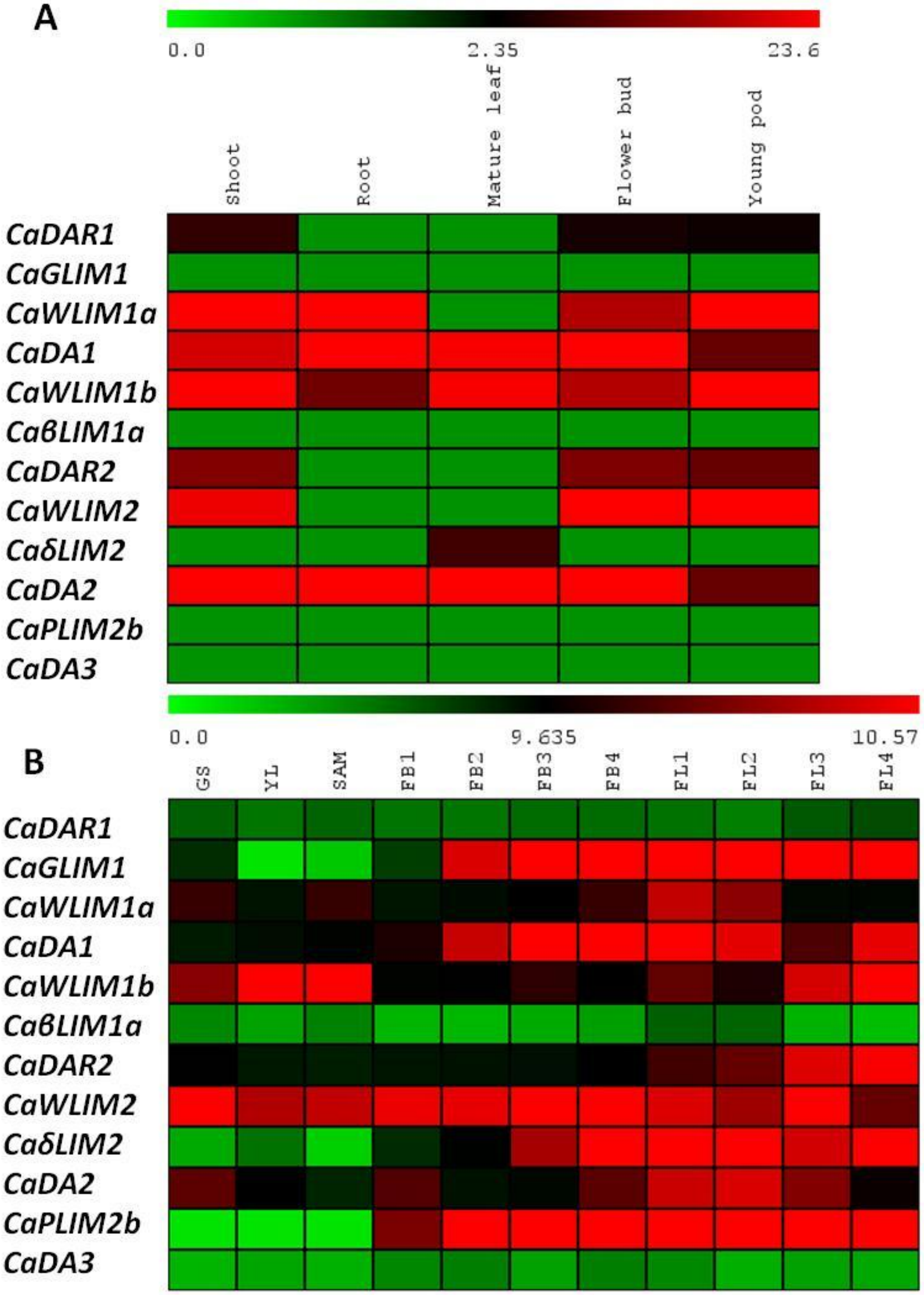

Supplement: S6 Fig — (A) Expression in shoots, roots, mature leaves, flower buds and young pods, generated by 454 pyrosequencing of cDNA libraries prepared from respective samples. (B) Expression in germinating seedling (GS), young leaf (YL), shoot apical meristem (SAM), flower bud stages (FB1-FB4) and flower stages (FL1-FL4) generated by Illumina sequencing of RNA-seq libraries prepared from respective samples. Green and red color gradients indicate lower or higher transcript abundance, respectively. (PDF) [file pone.0138719.s006.pdf]
